# Prognostic value of circulating glypican-4 in chronic heart failure

**DOI:** 10.1007/s00109-026-02667-9

**Published:** 2026-04-07

**Authors:** Nora Schwegel, Viktoria Höller, Viktoria Santner, David Kajetan Zach, Jakob Lugitsch, Axel Muendlein, Eva Maria Brandtner, Andreas Leiherer, Heinz Drexel, Arthur Mader, Andrea Borenich, Stefan Pilz, Martin Grübler, Markus Wallner, Klemens Ablasser, Ewald Kolesnik, Andreas Zirlik, Dirk von Lewinski, Nicolas Verheyen

**Affiliations:** 1 Present Address: Division of Cardiology, University Heart Center Graz, Medical University of Graz, Auenbruggerplatz 15, 8036 Graz, Austria; 2 Vorarlberg Institute for Vascular Investigation and Treatment (VIVIT), Feldkirch, Austria; 3 Institute for Medical Informatics, Statistics and Documentation, Medical University Graz, Graz, Austria; 4 Division of Endocrinology and Diabetology, Department of Internal Medicine, Medical University of Graz, Graz, Austria; 5 Department of Internal Medicine With Cardiology, Nephrology and Intensive Care Medicine, University Hospital Wiener Neustadt, Wiener Neustadt, Austria; 6 Medical Faculty, Sigmund Freud University, Vienna, Austria; 7 Department of Medicine, Faculty of Medicine and Dentistry, Danube Private University, Krems, Austria

**Keywords:** Chronic heart failure, Heart failure with reduced ejection fraction, Transthyretin amyloid cardiomyopathy, Glypican-4, Mortality

## Abstract

**Abstract:**

Glypican-4 (GPC-4), an endothelial cell surface protein, is released into the circulation in the context of ischemia, inflammation, neurohumoral activity, and shear stress. This study aimed to investigate the prognostic value of GPC-4 in chronic heart failure. GPC-4 concentrations were determined in two prospective cohorts: patients with chronic heart failure with reduced ejection fraction (HFrEF), and with transthyretin amyloid cardiomyopathy (ATTR-CM). Multivariable Cox regression analyses were adjusted for age, sex, estimated glomerular filtration rate, N-terminal B-type natriuretic peptide, and left ventricular ejection fraction. In HFrEF (*n* = 205, median age 66 years, 22% women), 58 patients (28%) died, 18 (9%) from cardiovascular cause, and 46 patients (22%) were hospitalized for worsening heart failure (WHF) during 4.2 years follow-up. In ATTR-CM (*n* = 121, median age 76 years, 12% women), 34 patients (28%) died, 12 (10%) from cardiovascular cause, and 32 patients (26%) had a WHF hospitalization during 2.2 years follow-up. Baseline GPC-4 (median [interquartile range]) levels were 1553 (1041, 1950) pg/ml in HFrEF, and 2071 (1579, 2893) pg/mL in ATTR-CM. In HFrEF, GPC-4 was independently associated with all-cause mortality (HR 1.69, 95%CI 1.22–2.32, *p* = 0.003) and cardiovascular mortality (HR 1.61, 95%CI 1.01–2.56, *p* = 0.045), but not with WHF hospitalizations. Conclusively, in ATTR-CM, GPC-4 independently predicted all-cause mortality (HR 1.96, 95%CI 1.12–3.44, *p* = 0.018), but not cardiovascular mortality and WHF hospitalizations. GPC-4 carries strong prognostic value for all-cause death in chronic heart failure across various etiologies, but not for heart-failure specific outcomes. Further studies are warranted to elucidate its value in cardiovascular disease.

**Key messages:**

- Glypican-4 rises with ischemia, inflammation, neurohumoral activity, and shear stress.
- Circulating glypican-4 predicts prognosis in chronic heart failure, regardless of cause.
- Glypican-4 predicted all-cause death in heart failure with reduced ejection fraction and restrictive cardiomyopathy, but not heart failure–specific outcomes.

**Supplementary Information:**

The online version contains supplementary material available at 10.1007/s00109-026-02667-9.

## Introduction

Chronic heart failure is one of the leading causes of mortality and hospitalizations worldwide, with an ever-increasing prevalence [1]. Despite advances in medical treatment, mortality remains high in patients with chronic heart failure, with reported 1-year mortality rates ranging from 8% up to 30% depending on the geographical region [1, 2]. Traditionally, clinical decisions and risk prediction are based on left ventricular ejection fraction and patient-reported symptoms [3]. Biomarkers are important in the diagnosis of heart failure, as well as in the assessment of prognosis and monitoring of treatment response. Novel biomarkers on top of established biomarkers such as natriuretic peptides are warranted to further improve risk prediction, especially in cohorts at particular risk, such as transthyretin amyloid cardiomyopathy (ATTR-CM) [4].

Glypicans are cell membrane bound heparan sulfate proteoglycans, which are involved in cellular proliferation and regulation of growth-factor signaling during development [5]. Glypican-4 (GPC-4), as a part of the glycocalyx covering the luminal surface of the endothelium, is a major factor for maintaining a healthy vasculature [6]. However, it is also found in a variety of other tissues, like visceral and subcutaneous adipose tissue, muscle tissue, in the placenta, lung, kidney, pancreas, and even the heart [7, 8]. GPC-4 is attached to the cell surface via a glycosylphosphatidylinositol (GPI) anchor, but it can be released into circulation by proteolytic shedding or GPI anchor cleavage, and the circulating domains of GPC-4 remain biologically active [9, 10]. The active release of GPC-4 into the bloodstream is linked to various clinical conditions, such as ischemia, inflammation, neurohumoral activation, and shear stress, besides others [8, 11–13]. GPC-4 is involved in the regulation of fibroblast growth factor, hepatocyte growth factor, Wnt, and bone morphogenic protein, potentially playing a major role in cardiac remodeling and fibrosis [14–17]. Circulating GPC-4 proved to be a valuable prognostic biomarker in different cardiovascular disorders like peripheral and coronary artery disease, and in acute heart failure [18–20].

However, data on the prognostic value of GPC-4 in patients with chronic heart failure is sparse, and previous studies did not consider known associations of GPC-4 with obesity, inflammation, and glucose metabolism. Furthermore, so far there is no data on GPC-4 in heart failure collectives with specific etiologies, and there is a lack of cut-off values of circulating GPC-4 for risk stratification in these patients [20]. This study aims to investigate the prognostic value of circulating GPC-4 in chronic heart failure, and to prove its applicability in heart failure cohorts of various etiologies. The predictive value of GPC-4 was investigated in two contemporary heart failure cohorts; patients with predominantly systolic dysfunction and patients with predominantly diastolic dysfunction.

## Material and methods

The present study is based on two prospective single-center cohorts including patients who were consecutively enrolled in the heart failure outpatient clinic of an academic tertiary referral center (Division of Cardiology, University Heart Center of Graz, Medical University of Graz, Graz, Austria).

Investigations on the prognostic value of GPC-4 were performed in the Role of Comorbidities in Chronic Heart Failure (RoC-HF) cohort. The methodological details and main in- and exclusion criteria of the RoC-HF study have been published previously [21]. In brief, it comprised 205 patients with a previous diagnosis of heart failure with reduced ejection fraction (HFrEF), with optimal medical therapy based on the Heart Failure guidelines 2016 of the European Society of Cardiology, and a left ventricular ejection fraction (LVEF) < 50% at inclusion [22].

In the second cohort, 121 patients were recruited from the local Hypertrophic Cardiomyopathy (HCM) Registry [23]. For the present study, participants with an established diagnosis of ATTR-CM and available blood sampling at the baseline visit were included. The diagnosis of ATTR-CM was made according to international recommendations [24].

All patients gave written informed consent for participation. Approval was granted by the local Ethics Committee (EC-No. 28–467 ex 15/16 and 30–286 ex 17/18) and the study was conducted in compliance with Good Clinical Practice, the Declaration of Helsinki, and the STROBE guidelines.

### Laboratory parameters

Blood samples were collected on the baseline visit in each patient. Routine laboratory parameters were immediately determined by standard laboratory methods at the Clinical Institute of Medical and Chemical Laboratory Diagnostics of the Medical University of Graz, Austria. N-terminal pro-brain natriuretic peptide (NT-proBNP) and interleukin-6 (IL-6) were measured using the electrochemiluminescence immunoassay (Roche Diagnostics, Switzerland) using an autoanalyser (Elecsys 2010). Serum creatinine was determined using the Jaffè assay (Roche Diagnostics, Switzerland), and estimated glomerular filtration rate (eGFR) was calculated according to the Chronic Kidney Disease Epidemiology Collaboration (CKD-EPI) equation. C-reactive protein (CRP) was measured using the immunoturbidimetry method from Roche Diagnostics (Roche Diagnostics, Switzerland). Hemoglobin A1c (HbA1c) was determined using high-performance liquid chromatography on an Adams HA-8180 V (A. Menarini Diagnostics, Florenz, Italy).

A prespecified volume of blood samples was centrifuged, aliquoted, and frozen at −80 °C at the local biobanking facility. Circulating GPC-4 levels were measured from one-time frozen serum at the Vorarlberg Institute for Vascular Investigation and Treatment (VIVIT, Feldkirch, Austria) using the commercial enzyme-linked immunosorbent assay (ELISA) kit of Cloude-Clone (Houston, Texas, USA; Article number: SEA998Hu) as described in detail in a previous publication [20]. Prior to analysis, serum samples were diluted 1:6 in phosphate buffered saline as a dilution buffer. Standardized working steps were performed according to the manual of the manufacturer. Measurement of the color change was performed at 450 nm using the BioTek 800TS Absorbance Reader (Agilent, Santa Clara, CA, USA), washing between steps was automatically performed by the BioTek 50 TS Washer (Agilent, Santa Clara, CA, USA). According to the manufacturers’ specifications, the assay kit had a detection range of 0.031–2.000 ng/mL, a minimum detectable dose of 0.013 ng/mL, an intra-assay coefficient of variation below 10%, and an inter-assay coefficient of variation below 12%.

### Follow-up

Patient outcomes were retrieved from local medical and health insurance records. In patients without documentation on the cause of death, telemedicine data of implanted cardiac devices were interrogated *post-mortem*, as available. Cause of death was adjudicated by an experienced cardiologist who was blinded to patients’ characteristics. If no data on the cause of death was available, death was classified as unknown. Hospitalization due to worsening heart failure (WHF) was defined as an unscheduled hospitalization due to documented heart failure signs and symptoms with 24 h of in-hospital stay and initiation or significant augmentation of heart failure therapy [25].

### Statistical analysis

Patient characteristics were described in the total patient samples and stratified by tertiles of GPC-4 levels. Continuous variables were described as median with interquartile range; categorical variables were expressed as count with percentage. Group comparisons were performed using Pearson’s chi-squared, Fisher’s exact, or Kruskal-Wallis rank sum tests, as appropriate. Correlations were assessed using Spearman correlation. Outcome associations were investigated using univariable Cox proportional hazard analyses and multivariable Cox-regression models employing baseline GPC-4 as an independent variable. Models were adjusted for established risk parameters in heart failure (age, sex, eGFR, NT-proBNP, LVEF) and further parameters (body mass index [BMI], IL-6, HbA1c) based on pathophysiological considerations. Patients were censored at the end of follow-up, the date of the outcome event, or the date of death, whatever occurred first. Competing risk analysis was performed using the Fine and Gray subdistribution hazard model and implemented in regression analyses with all-cause mortality as competing risk for cardiovascular mortality and WHF hospitalizations. Results were illustrated using Kaplan-Meier and cumulative incidence plots. To determine cut-off values, time-dependent receiver operating characteristic curves (ROC) and the according area under the curves (AUC) were calculated. Statistical analyses were performed using R (R statistical software, version 4.4.1), considering a *p*-value < 0.05 as statistically significant.

## Results

### Baseline characteristics

A total of 205 patients were enrolled in the HFrEF cohort, with a median (interquartile range) age of 66 (58, 74) years, 22% women, and a median LVEF of 37 (30, 43) %. The ATTR-CM cohort comprised 121 patients, with a median age of 79 (76, 82) years, 12% women, and a median LVEF of 52 (44, 59) %. In the HFrEF cohort GPC-4 levels were higher compared to the ATTR-CM cohort (1553 [1041, 1950] vs. 2071 [1579, 2893] pg/mL, *p* < 0.001). Detailed baseline characteristics of both cohorts are described in Table 1.

**Table 1:** Baseline characteristics

	All	Tertile 1	Tertile 2	Tertile 3	*p*	Available data, *n* (%)
HFrEF cohort, *n (%)*	205 (100)	69 (34)	68 (33)	68 (33)		
Glypican-4 tertiles, *pg/mL*		533–1165	1166–1766	1767–7156		
Age, *years*	66 (58, 74)	61 (56, 69)	64 (57, 73)	70 (66, 76)	< 0.001	205 (100)
Female sex, *n (%)*	45 (22)	10 (15)	20 (29)	15 (22)	0.108	205 (100)
Ethnicity Caucasian, *n (%)*	205 (100)	69 (100)	68 (100)	68 (100)	-	205 (100)
Body mass index, *kg/m*^*2*^	28.0 (25.2, 31.6)	27.5 (25.2, 30.1)	29.0 (25.5, 32.2)	28.5 (25.1, 32.6)	0.188	205 (100)
Systolic blood pressure, *mmHg*	120 (109, 134)	120 (108, 135)	121 (110, 134)	119 (109,132)	0.964	200 (98)
Diastolic blood pressure, *mmHg*	76 (68, 84)	77 (68, 84)	77 (69, 86)	75 (67, 82)	0.370	200 (98)
Heart rate, *bpm*	65 (59, 74)	62 (53, 71)	65 (59, 78)	67 (60, 74)	0.008	199 (97)
LVEF, *%*	37 (30, 43)	35 (30, 45)	36 (29, 42)	37 (31, 43)	0.473	205 (100)
NYHA-class, *n (%)*					0.170	205 (100)
II	138 (67)	53 (77)	42 (62)	43 (63)		
II–III	32 (16)	8 (12)	15 (22)	9 (13)		
III	33 (16)	7 (10)	11 (16)	15 (22)		
IV	2 (1)	1 (1)	0 (0)	1 (2)		
Angina pectoris symptoms, *n (%)*					0.240	201 (98)
Typical	18 (9)	7 (10)	7 (10)	4 (6)		
Atypical	19 (10)	3 (5)	10 (15)	6 (9)		
Comorbidities, *n (%)*						
Atrial fibrillation	85 (42)	16 (23)	31 (46)	38 (56)	< 0.001	205 (100)
Coronary artery disease	143 (70)	44 (64)	47 (69)	52 (77)	0.267	205 (100)
Hypertension	143 (73)	42 (64)	55 (82)	46 (73)	0.076	196 (96)
Diabetes mellitus	63 (31)	10 (14)	21 (31)	32 (47)	< 0.001	205 (100)
Chronic kidney disease	77 (38)	15 (22)	22 (33)	40 (59)	< 0.001	196 (96)
Laboratory parameters						
Glypican-4, *pg/mL*	1553 (1041, 1950)	900 (773, 1041)	1557 (1368, 1649)	2405 (1950, 3354)	< 0.001	205 (100)
NT-proBNP, *pg/mL*	964 (336, 2151)	456 (218, 974)	1002 (379, 2518)	1650 (830, 3328)	< 0.001	205 (100)
Estimated GFR, *mL/min/1.73 m*^*2*^	64 (48, 79)	82 (71, 96)	65 (52, 76)	46 (34, 55)	< 0.001	199 (97)
Creatinine, *mg/dL*	1.1 (0.9, 1.4)	0.9 (0.8, 1.1)	1.1 (1.0, 1.3)	1.4 (1.2, 2.0)	< 0.001	199 (97)
CRP, *mg/L*	2 (1, 5)	2 (1, 3)	3 (1, 5)	4 (1, 9)	< 0.001	197 (96)
Interleukin-6, *pg/mL*	5 (3, 8)	3 (2, 5)	5 (3, 7)	7 (5, 11)	< 0.001	198 (97)
HbA1c, *mmol/mol*	40 (37, 46)	39 (37, 42)	42 (38, 47)	40 (37, 53)	0.031	198 (97)
ATTR-CM cohort, *n (%)*	121 (100)	41 (34)	40 (33)	40 (33)		
Glypican-4 tertiles, *pg/mL*		948–1761	1762–2717	2718–5995		
Age, *years*	79 (76, 82)	76 (71, 80)	80 (77, 82)	80 (78, 83)	< 0.001	121 (100)
Female sex, *n (%)*	14 (12)	4 (10)	5 (13)	5 (13)	0.881	121 (100)
Ethnicity Caucasian, *n (%)*	121 (100)	41 (100)	40 (100)	40 (100)	-	121 (100)
Body mass index, *kg/m*^*2*^	25.3 (23.1, 27.3)	24.9 (22.6, 26.5)	25.7 (23.5, 27.2)	25.5 (22.9, 28.6)	0.547	106 (88)
Systolic blood pressure, *mmHg*	132 (119, 148)	138 (121, 158)	132 (119, 150)	126 (118, 141)	0.058	100 (83)
Diastolic blood pressure, *mmHg*	78 (71, 85)	79 (71, 84)	82 (74, 88)	76 (66, 82)	0.052	100 (83)
Heart rate, *bpm*	71 (61, 80)	66 (60, 80)	73 (62, 80)	72 (64, 79)	0.528	86 (72)
LVEF, *%*	52 (44, 59)	53 (48, 60)	51 (43, 58)	50 (42, 57)	0.340	118 (98)
NYHA-class, *n (%)*					0.013	105 (87)
I	13 (12)	7 (18)	3 (8)	3 (10)		
II	23 (22)	15 (39)	4 (11)	4 (13)		
II–III	26 (25)	9 (24)	11 (31)	6 (19)		
III	39 (37)	6 (16)	17 (47)	16 (52)		
IV	4 (4)	1 (3)	1 (3)	2 (7)		
Angina pectoris symptoms, *n (%)*					0.554	106 (88)
Typical	19 (18)	5 (13)	8 (22)	6 (19)		
Atypical	12 (11)	5 (13)	2 (5)	5 (16)		
Comorbidities, *n (%)*						
Atrial fibrillation	60 (58)	13 (35)	24 (65)	23 (77)	0.002	103 (86)
Coronary artery disease	48 (45)	10 (26)	17 (46)	21 (68)	0.017	109 (88)
Hypertension	76 (63)	26 (63)	26 (65)	24 (60)	0.795	104 (86)
Diabetes mellitus	23 (22)	7 (18)	11 (30)	5 (16)	0.331	106 (88)
Chronic kidney disease	49 (48)	9 (24)	18 (51)	22 (73)	< 0.001	102 (85)
Laboratory parameters						
Glypican-4, *pg/mL*	2071 (1579, 2893)	1481 (1297, 1579)	2075 (1986, 2331)	3240 (2898, 3842)	< 0.001	117 (97)
NT-proBNP, *pg/mL*	2673 (1412, 5033)	1582 (930, 2611)	2691 (1400, 4586)	4644 (2822, 6955)	< 0.001	112 (94)
Estimated GFR, *mL/min/1.73 m*^*2*^	58 (46, 69)	70(61, 81)	58 (47, 64)	43 (34, 52)	< 0.001	112 (94)
Creatinine, *mg/dL*	1.2 (1.0, 1.5)	1.0 (0.9, 1.1)	1.2 (1.0, 1.4)	1.5 (1.3, 1.8)	< 0.001	112 (94)
CRP, *mg/L*	2 (1, 4)	1 (1, 2)	3 (1, 4)	4 (2, 10)	< 0.001	112 (94)
Interleukin-6, *pg/mL*	5 (3, 10)	3 (2, 5)	5 (3, 10)	9 (5, 14)	< 0.001	110 (92)
HbA1c,* mmol/mol*	41 (38, 45)	40 (37, 44)	43 (39, 47)	41 (39, 44)	0.246	108 (90)

All parameters reported in median (interquartile range) or frequency (percentage). *p*-values derived from Pearson’s chi-squared, Fisher’s exact, or Kruskal-Wallis rank sum tests, as appropriate. *Obesity is defined according to the World Health Organization (WHO) as BMI ≥ 30 kg/m^2^. *HFrEF* heart failure with reduced ejection fraction, *LVEF* left ventricular ejection fraction, *NYHA* New York Heart Association, *NT-proBNP* N-terminal pro-brain natriuretic peptide, *GFR* glomerular filtration rate, *CRP* C-reactive protein, *HbA1c* hemoglobin A1c, *ATTR-CM* transthyretin amyloid cardiomyopathy

In the HFrEF cohort, parameters associated with GPC-4 levels were age, NT-proBNP, eGFR, CRP, IL-6, and HbA1c. Similar associations were found in the ATTR-CM cohort. Both are described in detail in Table 2.

**Table 2:** Correlation analyses

	HFrEF cohort		ATTR-CM cohort	
GPC-4	Spearman *r*	*p*-value	Spearman*** r***	*p*-value
Age	0.34	< 0.001	0.30	< 0.001
eGFR	−0.72	< 0.001	−0.71	< 0.001
NT-proBNP	0.46	< 0.001	0.57	< 0.001
LVEF	−0.01	0.834	−0.14	0.141
BMI	0.12	0.093	0.15	0.138
IL-6	0.50	< 0.001	0.55	< 0.001
HbA1c	0.19	0.007	0.07	0.467

Correlation between glypican-4 and clinical variables, presented as Spearman *r *and according *p*-value. *HFrEF* heart failure with reduced ejection fraction, *ATTR-CM* transthyretin amyloid cardiomyopathy, *GPC-4* glypican-4, *eGFR* estimated glomerular filtration rate, *NT-proBNP* N-terminal pro-brain natriuretic peptide, *LVEF* left ventricular ejection fraction, *BMI* body mass index, *IL-6* interleukin-6, *HbA1c* hemoglobin A1c

### Outcome analyses in the HFrEF cohort

**Fig. 1:**
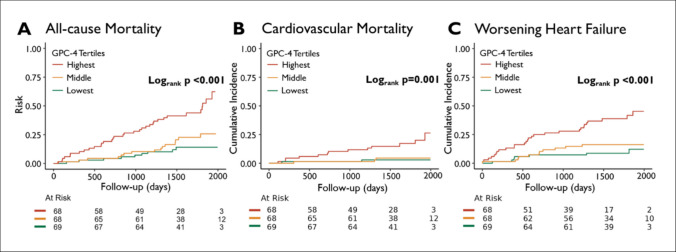
Kaplan-Meier estimates and cumulative incidence plots according to tertiles of GPC-4 in the HFrEF cohort. **A** Kaplan-Meier curves for all-cause mortality and cumulative incidence plots for **B** cardiovascular mortality and **C** worsening heart failure hospitalization in the HFrEF cohort, stratified by tertiles of GPC-4 (highest 1767–7156 pg/mL; middle 1166–1766 pg/mL; lowest 533–1165 pg/mL). *p*-values were obtained from log-rank tests. *GPC-4* glypican 4, *HFrEF* heart failure with reduced ejection fraction

In the HFrEF cohort, with a median follow-up of 4.2 (3.6, 5.1) years, 58 patients (28%) died, 18 patients (9%) died of cardiovascular cause, and 46 patients (22%) were hospitalized due to WHF. Figure 1 represents the Kaplan-Meier analyses and cumulative incidence plots stratified by tertiles of GPC-4, which illustrates a graded increased risk with increased GPC-4 levels. Univariable regression analyses indicated an increased risk of all-cause mortality (HR 1.92, 95%CI 1.59–2.33, *p* < 0.001), cardiovascular mortality (HR 2.10, 95%CI 1.58–2.79, *p* < 0.001), and WHF hospitalizations (HR 1.57, 95%CI 1.28–1.94, *p* < 0.001). These associations remained significant after adjusting for age and sex for all clinical endpoints (model 1; *p* < 0.001 each; Table 3). After further adjustment for eGFR, NT-proBNP, and LVEF (model 2), GPC-4 levels remained significantly associated with a higher risk of all-cause mortality (HR 1.69, 95%CI 1.22–2.32, *p* = 0.003) and cardiovascular mortality (HR 1.61, 95%CI 1.01–2.56, *p* = 0.045) but the association with risk of WHF hospitalizations ceased to be significant (HR 1.15, 95%CI 0.75–1.76, *p* = 0.515). Regarding the increased risk of all-cause mortality with increased GPC-4 levels, the association remained significant after additional adjustment for body mass index (BMI) (model 3; HR 1.66, 95%CI 1.20–2.29, *p* = 0.004), or IL-6 (model 4; HR 1.55, 95%CI 1.13–2.13, *p* = 0.009), or HbA1c (model 5; HR 1.66, 95%CI 1.19–2.30, *p* = 0.004). Full analyses are provided in *Table S1*.

**Table 3:** Outcome analyses

Glypican-4 per SD increase	All-cause mortality			Cardiovascular mortality			Worsening heart failure hospitalization		
	HR	95% CI	*p*-value	HR	95% CI	*p*-value	HR	95% CI	*p*-value
HFrEF cohort									
Univariate	1.92	1.59, 2.33	< 0.001	2.10	1.58, 2.79	< 0.001	1.57	1.28, 1.94	< 0.001
Model 1	1.84	1.52, 2.23	< 0.001	1.93	1.45, 2.57	< 0.001	1.50	1.21, 1.86	< 0.001
Model 2	1.69	1.22, 2.32	0.003	1.61	1.01, 2.56	0.045	1.15	0.75, 1.76	0.515
Model 3	1.66	1.20, 2.29	0.004						
Model 4	1.55	1.13, 2.13	0.009						
Model 5	1.66	1.19, 2.30	0.004						
ATTR-CM cohort									
Univariate	2.05	1.47, 2.86	< 0.001	1.49	0.81, 2.72	0.198	1.65	1.22, 2.23	0.001
Model 1	2.15	1.48, 3.12	< 0.001				1.69	1.20, 2.38	0.003
Model 2	1.96	1.12, 3.44	0.018				0.89	0.50, 1.58	0.681
Model 3	1.93	1.05, 3.57	0.035						
Model 4	2.04	1.15, 3.63	0.013						
Model 5	1.96	1.13, 3.41	0.016						

Results from Cox proportional hazards regression analyses, including competing risk analysis according to the Fine and Gray model. Presented as hazard ratios (HR) and 95% confidence intervals (CI) per one standard deviation (SD) increase of glypican-4. Multivariable analyses adjusted for age and sex (model 1); further adjusted for estimated glomerular filtration rate, N-terminal pro-brain natriuretic peptide, and left ventricular ejection fraction (model 2); and additionally adjusted for either body mass index (model 3); or interleukin-6 (model 4); or hemoglobin A1c (model 5). *HFrEF* heart failure with reduced ejection fraction, *ATTR-CM* transthyretin amyloid cardiomyopathy

### Outcome analyses in the ATTR-CM cohort

**Fig. 2:**
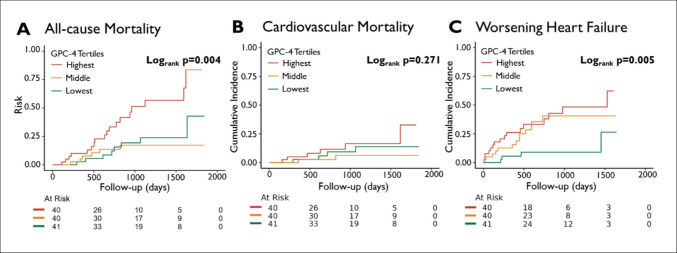
Kaplan-Meier estimates and cumulative incidence plots according to tertiles of GPC-4 in the ATTR-CM cohort. **A** Kaplan-Meier curves for all-cause mortality and cumulative incidence plots for **B** cardiovascular mortality and **C** worsening heart failure hospitalization in the ATTR-CM cohort, stratified by tertiles of GPC-4 (highest 2718–5995 pg/mL; middle 1762–2717 pg/mL; lowest 948–1761 pg/mL). *p*-values were obtained from log-rank tests. *GPC-4* glypican 4, *ATTR-CM* transthyretin amyloid cardiomyopathy

In ATTR-CM, with a median follow-up of 2.2 (1.3, 3.3) years, 34 patients (28%) died, 12 patients (10%) died of cardiovascular cause, and 32 patients (26%) had a WHF hospitalization. The Kaplan-Meier analyses and cumulative incidence plots are illustrated in Fig. 2. In univariable Cox-regression GPC-4 levels were associated with a higher risk of all-cause mortality (HR 2.05, 95%CI 1.47–2.86, *p* < 0.001) and WHF hospitalizations (HR 1.65, 95%CI 1.22–2.23, *p* = 0.001); no significant association to cardiovascular mortality was observed with competing risk analysis (HR 1.49, 95%CI 0.81–2.72, *p* = 0.198). The associations with all-cause mortality and WHF hospitalizations remained significant after adjustment for age and sex (model 1; all-cause mortality HR 2.15, 95%CI 1.48–3.12, *p* < 0.001; WHF hospitalizations HR 1.69, 95%CI 1.20–2.38, *p* = 0.003; Table 3). In model 2, further adjusted for eGFR, NT-proBNP, and LVEF, elevated GPC-4 levels indicated an increased risk for all-cause mortality (HR 1.96, 95%CI 1.12–3.44, *p* = 0.018), but the association with WHF hospitalizations became neutral (HR 0.89, 95%CI 0.50–1.58, *p* = 0.681). The association of elevated GPC-4 levels and increased risk for all-cause mortality remained significant after further adjustment for BMI (model 3; HR 1.93, 95%CI 1.05–3.57, *p* = 0.035), or IL-6 (model 4; HR 2.04, 95%CI 1.15–3.63, *p* = 0.013), or HbA1c (model 5; HR 1.96, 95%CI 1.13–3.41, *p* = 0.016), with comparable albeit slightly higher hazard ratios compared to the HFrEF cohort. Full analyses are provided in *Table S2*.

### AUC analyses

Results from ROC-AUC analyses, maximizing the balance between specificity and sensitivity, suggest a serum level of GPC-4 at 1630 pg/mL to be the best cut-off value for risk stratification for all-cause mortality in HFrEF. In ATTR-CM, with overall higher GPC-4 levels, the best cut-off was 2774 pg/mL. Endpoint-specific analyses and ROC-AUC analyses are provided in the Supplementary Table S3 and Figure S1–2.

## Discussion

In this study, serum concentrations of GPC-4 were independently associated with an increased risk for all-cause mortality in patients with chronic heart failure, even after adjustment for important confounders including natriuretic peptides. Furthermore, this is the first study to investigate GPC-4 in cardiac amyloidosis. However, GPC-4 was not independently predictive of heart failure–specific outcomes (cardiovascular death and WHF hospitalizations). This is the first study reporting on associations between GPC-4 concentrations and cardiovascular outcomes in stable outpatients with chronic heart failure.

Our results are in line with previous studies and significantly add to the limited data on GPC-4 in heart failure patients. Similar associations with clinical outcomes were described in a large cohort of patients referred to elective coronary angiography for evaluation of established or suspected coronary artery disease. Here, GPC-4 was independently associated with an increased risk of major adverse cardiovascular events and all-cause mortality independently of traditional cardiovascular risk factors [19]. This association with clinical outcomes was also demonstrated by the same working group in patients with peripheral artery disease as well as in a cohort of patients with heart failure [18, 20]. Muendlein and colleagues’ cohort comprised patients with heart failure across the whole spectrum of ejection fraction and 80% met the criteria for acute heart failure. In contrast to this prior study focusing largely on an acute heart failure population, the present analysis investigated GPC-4 as a prognostic biomarker specifically in a contemporary outpatient clinic cohort with chronic and stable heart failure, highlighting its potential utility for long-term risk assessment in outpatient care. In the present study, GPC-4 was associated with a higher risk of all-cause mortality and was an even superior prognosticator compared to NT-proBNP. Emerging evidence suggests that GPC-4 provides prognostic information that is independent of and additive to established biomarkers like NT-proBNP [19, 20]. Our study not only confirms these findings in a contemporary cohort of patients with stable chronic heart failure with predominantly systolic dysfunction but also shows that circulating GPC-4 predicts all-cause mortality in patients with ATTR-CM, a restrictive cardiomyopathy with predominantly diastolic dysfunction.

Regardless of etiology, comorbidities including chronic kidney disease, inflammation, impaired glucose tolerance, and insulin resistance are common in patients with heart failure and have a strong impact on prognosis [21]. The correlation and interaction between GPC-4 and parameters reflecting these pathologies deserve some discussion. GPC-4 directly interacts with the insulin receptor, enhances receptor signaling, and enhances adipose cell differentiation [10]. These functions are independent of its GPI anchorage; therefore also the circulating domain of GPC-4 acts as an insulin-sensitizing adipokine. Ussar et al. [10] showed that serum levels of GPC-4 are positively correlated with both body fat content and insulin resistance. Several other studies also found significantly increased levels in patients with insulin resistance and metabolic syndrome. Here, GPC-4 levels correlated with fasting blood glucose, fasting insulin, the Homeostatic Model Assessment for Insulin Resistance (HOMA-IR) score, and arterial stiffness, which are useful markers for assessing cardiovascular risk related to metabolic disease [26, 27]. Furthermore, as a component of the endothelial glycocalyx, GPC-4 also plays a major part in maintaining the integrity of the vasculature surface. This protects cells from oxidants, immune cells, and cytokines. Shedding and degradation of the glycocalyx are promoted in several age-related cardiovascular diseases and inflammatory conditions [6]. Fisher et al. [28] showed increased serum GPC-4 levels in patients with sepsis. The most likely source of these elevated GPC-4 serum levels seems to be extensive shedding of the endothelial glycocalyx. However, GPC-4 is also expressed in monocytes and fibrocytes, but whether shedding from these cells in response to inflammation and cell stress contributes to elevated serum levels has yet to be investigated. As systemic inflammation is a common pathologic feature of chronic heart failure and is linked to disease development, progression, and outcome it can be assumed that increased GPC-4 levels are at least partly the result of endothelial glycocalyx damage due to inflammatory conditions [29].

Our present observations are concordant with these prior reports, demonstrating a strong association between GPC-4 and kidney function, obesity, glucose metabolism, and inflammation. The putative presence of comorbidities might have contributed to elevated levels of GPC-4 and exposed the patients to an increased risk a priori. However, in multivariable Cox regression models adjusted for these parameters, associations between GPC-4 and all-cause mortality remained significant. This leads to the assumption that these pathways may have contributed to the association between elevated GPC-4 levels and a worse outcome in patients with heart failure but cannot solely explain this association. Interestingly, the association between GPC-4 and heart failure–specific outcomes (cardiovascular death, WHF hospitalizations) was non-significant in multivariate models. Based on pathophysiological considerations, it may be speculated that GPC-4 is rather a function of vascular than of cardiac disease and its best merit as a prognostic biomarker may lie in the field of vascular diseases.

An association between the prognostic value of GPC-4 serum levels for clinical outcomes and genetic alterations seems possible. However, so far pathogenic genetic variability and single nucleotide polymorphisms affecting GPC-4 levels have only been described in the context of Keipert Syndrome and behavioral alterations [30]. A possible protein-cardiometabolic trait relationship should be addressed in genome-wide association studies.

## Strengths and limitations

Some drawbacks of our study deserve mentioning. The observational design does not allow definite conclusions on causality of the association between GPC-4 and clinical outcomes, nor on the underlying pathophysiological mechanisms. Another limitation is the single-center design which limits the generalizability of observed results. A key methodological limitation is that GPC-4 levels measured in our cohort cannot be directly compared to levels reported for healthy populations. Circulating protein levels are strongly influenced by assay-specific factors, limiting cross-study comparability. Accordingly, our analyses were restricted to relative, within-cohort comparisons.

The enrollment of a rather frail population from a large catchment area, especially in the ATTR-CM cohort, results in a relatively high proportion of unclassified deaths in this cohort. This leads to a limited generalizability and significance of the observed results regarding cardiovascular mortality.

Strengths include a comprehensive adjustment in regression models for important confounding parameters. Statistical models addressed the most relevant pathways impacting the circulating levels of GPC-4, namely obesity, impaired glucose tolerance, and inflammation.

Literature indicates different levels of GPC-4 by gender. However, we did not find a statistically significant difference in our cohorts. The relevance of this finding in the ATTR-CM cohort is limited due to the low proportion of women in this group. However, this is a common limitation in ATTR-CM-related studies due to the predominantly male prevalence of this disease.

## Conclusion

GPC-4 carries strong prognostic value for all-cause death in chronic heart failure across various etiologies but is not independently associated with cardiovascular death and WHF hospitalization as heart failure–specific outcomes. Further studies are warranted to elucidate the value of GPC-4 in cardiovascular disease.

## Supplementary Information

Below is the link to the electronic supplementary material.

ESM 1 DOCX (219 KB)

## Data Availability

The data that support the findings of this study are available from the corresponding author upon reasonable request.

## Abbreviations

AUC: Area under the curves
ATTR-CM: Transthyretin amyloid cardiomyopathy
BMI: Body mass index
CI: Confidence interval
CRP: C-reactive protein
eGFR: Estimated glomerular filtration rate
ELISA: Enzyme-linked immunosorbent assay
GPC-4: Glypican-4
GPI: Glycosylphosphatidylinositol
HbA1c: Hemoglobin A1c
HCM: Hypertrophic cardiomyopathy
HFrEF: Heart failure with reduced ejection fraction
HR: Hazard ratio
IL-6: Interleukin-6
LVEF: Left ventricular ejection fraction
NT-proBNP: N-terminal pro-brain natriuretic peptide
ROC: Receiver operating characteristic curves
RoC-HF: Role of Comorbidities in Chronic Heart Failure
WHF: Worsening heart failure

## References

[1] Tsao CW, Aday AW, Almarzooq ZI, Anderson CAM, Arora P, Avery CL et al (2023) Heart disease and stroke statistics-2023 update: a report from the American Heart Association. Circulation 147(8):e93–e621 36695182 10.1161/CIR.0000000000001123 PMC12135016

[2] Crespo-Leiro MG, Anker SD, Maggioni AP, Coats AJ, Filippatos G, Ruschitzka F et al (2016) European Society of Cardiology Heart Failure Long-Term Registry (ESC-HF-LT): 1-year follow-up outcomes and differences across regions. Eur J Heart Fail 18(6):613–625 27324686 10.1002/ejhf.566

[3] McDonagh TA, Metra M, Adamo M, Gardner RS, Baumbach A, Bohm M et al (2021) 2021 ESC guidelines for the diagnosis and treatment of acute and chronic heart failure. Eur Heart J 42(36):3599–3726 34447992 10.1093/eurheartj/ehab368

[4] Berthelot E, Broussier A, Hittinger L, Donadio C, Rovani X, Salengro E et al (2023) Patients with cardiac amyloidosis are at a greater risk of mortality and hospital readmission after acute heart failure. ESC Heart Fail 10(3):2042–50 37051755 10.1002/ehf2.14337 PMC10192232

[5] Tumova S, Woods A, Couchman JR (2000) Heparan sulfate proteoglycans on the cell surface: versatile coordinators of cellular functions. Int J Biochem Cell Biol 32(3):269–288 10716625 10.1016/s1357-2725(99)00116-8

[6] Machin DR, Phuong TT, Donato AJ (2019) The role of the endothelial glycocalyx in advanced age and cardiovascular disease. Curr Opin Pharmacol 45:66–71 31112922 10.1016/j.coph.2019.04.011 PMC7055464

[7] Yoo HJ, Hwang SY, Cho GJ, Hong HC, Choi HY, Hwang TG et al (2013) Association of glypican-4 with body fat distribution, insulin resistance, and nonalcoholic fatty liver disease. J Clin Endocrinol Metab 98(7):2897–901 23633195 10.1210/jc.2012-4297

[8] Urschel K, Hug KP, Zuo H, Buttner M, Furtmair R, Kuehn C et al (2023) The shear stress-regulated expression of Glypican-4 in endothelial dysfunction in vitro and its clinical significance in atherosclerosis. Int J Mol Sci. 10.3390/ijms241411595 37511353 10.3390/ijms241411595 PMC10380765

[9] Huang K, Park S (2021) Heparan sulfated glypican-4 is released from astrocytes by proteolytic shedding and GPI-anchor cleavage mechanisms. eNeuro. 10.1523/ENEURO.0069-21.2021 34301723 10.1523/ENEURO.0069-21.2021 PMC8387153

[10] Ussar S, Bezy O, Bluher M, Kahn CR (2012) Glypican-4 enhances insulin signaling via interaction with the insulin receptor and serves as a novel adipokine. Diabetes 61(9):2289–2298 22751693 10.2337/db11-1395 PMC3425403

[11] Rehm M, Bruegger D, Christ F, Conzen P, Thiel M, Jacob M et al (2007) Shedding of the endothelial glycocalyx in patients undergoing major vascular surgery with global and regional ischemia. Circulation 116(17):1896–906 17923576 10.1161/CIRCULATIONAHA.106.684852

[12] Kim YH, Nijst P, Kiefer K, Tang WH (2017) Endothelial glycocalyx as biomarker for cardiovascular diseases: mechanistic and clinical implications. Curr Heart Fail Rep 14(2):117–126 28233259 10.1007/s11897-017-0320-5 PMC5386320

[13] Christensen G, Herum KM, Lunde IG (2019) Sweet, yet underappreciated: Proteoglycans and extracellular matrix remodeling in heart disease. Matrix Biol 75–76:286–99 29337052 10.1016/j.matbio.2018.01.001

[14] Strate I, Tessadori F, Bakkers J (2015) Glypican4 promotes cardiac specification and differentiation by attenuating canonical Wnt and Bmp signaling. Development 142(10):1767–1776 25968312 10.1242/dev.113894

[15] MacLean J, Pasumarthi KB (2014) Signaling mechanisms regulating fibroblast activation, phenoconversion and fibrosis in the heart. Indian J Biochem Biophys 51(6):476–482 25823219

[16] Mungunsukh O, McCart EA, Day RM (2014) Hepatocyte growth factor isoforms in tissue repair, cancer, and fibrotic remodeling. Biomedicines 2(4):301–326 28548073 10.3390/biomedicines2040301 PMC5344272

[17] Bertaud A, Joshkon A, Heim X, Bachelier R, Bardin N, Leroyer AS et al (2023) Signaling pathways and potential therapeutic strategies in cardiac fibrosis. Int J Mol Sci. 10.3390/ijms24021756 36675283 10.3390/ijms24021756 PMC9866199

[18] Muendlein A, Heinzle C, Leiherer A, Geiger K, Brandtner EM, Gaenger S et al (2022) Serum glypican-4 is associated with the 10-year clinical outcome of patients with peripheral artery disease. Int J Cardiol 369:54–9 35944770 10.1016/j.ijcard.2022.08.018

[19] Muendlein A, Brandtner EM, Leiherer A, Geiger K, Heinzle C, Gaenger S et al (2022) Serum glypican-4 is a marker of future vascular risk and mortality in coronary angiography patients. Atherosclerosis 345:33–38 35202959 10.1016/j.atherosclerosis.2022.02.015

[20] Muendlein A, Heinzle C, Leiherer A, Brandtner EM, Geiger K, Gaenger S et al (2023) Circulating glypican-4 is a new predictor of all-cause mortality in patients with heart failure. Clin Biochem 121–122:110675 37844682 10.1016/j.clinbiochem.2023.110675

[21] Verheyen N, Schmid J, Kolesnik E, Schwegel N, Spath J, Kattnig L et al (2024) Prevalence and prognostic impact of bone disease in chronic heart failure with reduced ejection fraction. ESC Heart Fail 11(3):1730–8 38450879 10.1002/ehf2.14741 PMC11098648

[22] Ponikowski P, Voors AA, Anker SD, Bueno H, Cleland JGF, Coats AJS et al (2016) 2016 ESC guidelines for the diagnosis and treatment of acute and chronic heart failure: the task force for the diagnosis and treatment of acute and chronic heart failure of the European Society of Cardiology (ESC)developed with the special contribution of the Heart Failure Association (HFA) of the ESC. Eur Heart J 37(27):2129–2200 27206819 10.1093/eurheartj/ehw128

[23] Holler V, Seebacher H, Zach D, Schwegel N, Ablasser K, Kolesnik E et al (2021) Myocardial deformation analysis in MYBPC3 and MYH7 related sarcomeric hypertrophic cardiomyopathy-the Graz Hypertrophic Cardiomyopathy Registry. Genes (Basel). 10.3390/genes12101469 34680864 10.3390/genes12101469 PMC8535960

[24] Garcia-Pavia P, Rapezzi C, Adler Y, Arad M, Basso C, Brucato A et al (2021) Diagnosis and treatment of cardiac amyloidosis: a position statement of the ESC Working Group on Myocardial and Pericardial Diseases. Eur Heart J 42(16):1554–68 33825853 10.1093/eurheartj/ehab072 PMC8060056

[25] Hicks KA, Mahaffey KW, Mehran R, Nissen SE, Wiviott SD, Dunn B et al (2018) 2017 cardiovascular and stroke endpoint definitions for clinical trials. Circulation 137(9):961–972 29483172 10.1161/CIRCULATIONAHA.117.033502

[26] Zhang K, Zhu H, Wang L, Yang H, Pan H, Gong F (2021) Serum glypican4 and glycosylphosphatidylinositol-specific phospholipase D levels are associated with adipose tissue insulin resistance in obese subjects with different glucose metabolism status. J Endocrinol Invest 44(4):781–790 32816247 10.1007/s40618-020-01372-9

[27] Li K, Xu X, Hu W, Li M, Yang M, Wang Y et al (2014) Glypican-4 is increased in human subjects with impaired glucose tolerance and decreased in patients with newly diagnosed type 2 diabetes. Acta Diabetol 51(6):981–90 25240528 10.1007/s00592-014-0652-5

[28] Fisher J, Linder A, Bentzer P (2019) Elevated plasma glypicans are associated with organ failure in patients with infection. Intensive Care Med Exp 7(1):2 30618011 10.1186/s40635-018-0216-z PMC6323058

[29] Rauchhaus M, Doehner W, Francis DP, Davos C, Kemp M, Liebenthal C et al (2000) Plasma cytokine parameters and mortality in patients with chronic heart failure. Circulation 102(25):3060–3067 11120695 10.1161/01.cir.102.25.3060

[30] Veugelers M, Vermeesch J, Watanabe K, Yamaguchi Y, Marynen P, David G (1998) GPC4, the gene for human K-glypican, flanks GPC3 on xq26: deletion of the GPC3-GPC4 gene cluster in one family with Simpson-Golabi-Behmel syndrome. Genomics 53(1):1–11 9787072 10.1006/geno.1998.5465

