# Supplementary material for: Prognostic value of circulating glypican-4 in chronic heart failure
Source: J Mol Med (Berl). 2026 Apr 7;104(1):62. doi: 10.1007/s00109-026-02667-9 (PMC13056786; doi:10.1007/s00109-026-02667-9)
Supplement: Supplementary file 1 — DOCX (219 KB) [file 109_2026_2667_MOESM1_ESM.docx]

***Supplementary Material***

**Prognostic value of circulating glypican-4 in chronic heart failure**

Nora Schwegel^1^, Viktoria Höller^1^, Viktoria Santner^1^, David Kajetan Zach^1^, Jakob Lugitsch^1^, Axel Muendlein^2^, Eva Maria Brandtner^2^, Andreas Leiherer^2^, Heinz Drexel^2^, Arthur Mader^2^, Andrea Borenich^3^, Stefan Pilz^4^, Martin Grübler^5,6,7^, Markus Wallner^1^, Klemens Ablasser^1^, Ewald Kolesnik^1^, Andreas Zirlik^1^, Dirk von Lewinski^1#^ , Nicolas Verheyen^1#*^

1 Division of Cardiology, University Heart Center, Medical University of Graz, Graz, Austria

2 Vorarlberg Institute for Vascular Investigation and Treatment (VIVIT), Feldkirch, Austria

3 Institute for Medical Informatics, Statistics and Documentation, Medical University Graz, Graz, Austria

4 Division of Endocrinology and Diabetology, Department of Internal Medicine, Medical University of Graz, Graz, Austria

5 Department of Internal Medicine with Cardiology, Nephrology and Intensive Care Medicine, University Hospital Wiener Neustadt, Wiener Neustadt, Austria

6 Medical Faculty, Sigmund Freud University, Vienna, Austria

7 Department of Medicine, Faculty of Medicine and Dentistry, Danube Private University, Krems, Austria

**Supplementary Table S1.** Cox proportional hazard analyses in the HFrEF cohort.

|  | **univariable** | | | **Model 1** | | | **Model 2** | | | **Model 3** | | | **Model 4** | | | **Model 5** | | |
| --- | --- | --- | --- | --- | --- | --- | --- | --- | --- | --- | --- | --- | --- | --- | --- | --- | --- | --- |
|  | **HR** | **95%CI** | **p** | **HR** | **95%CI** | **p** | **HR** | **95%CI** | **p** | **HR** | **95%CI** | **p** | **HR** | **95%CI** | **p** | **HR** | **95%CI** | **p** |
| **All-cause mortality** | | | | | | | | | |  |  |  |  |  |  |  |  |  |
| GPC-4 | 1.92 | 1.59, 2.33 | <0.001 | 1.84 | 1.52, 2.23 | <0.001 | 1.69 | 1.22, 2.32 | 0.003 | 1.66 | 1.20, 2.29 | 0.004 | 1.55 | 1.13, 2.13 | 0.009 | 1.66 | 1.19, 2.30 | 0.004 |
| Age |  |  |  | 1.03 | 1.00, 1.06 | 0.047 | 1.03 | 1.00, 1.07 | 0.062 | 1.04 | 1.00, 1.07 | 0.041 | 1.02 | 0.99, 1.06 | 0.166 | 1.04 | 1.00, 1.07 | 0.049 |
| Sex, female |  |  |  | 0.22 | 0.08, 0.60 | <0.001 | 0.18 | 0.06, 0.53 | <0.001 | 0.16 | 0.05, 0.49 | <0.001 | 0.14 | 0.04, 0.46 | <0.001 | 0.17 | 0.06, 0.51 | <0.001 |
| eGFR |  |  |  |  |  |  | 0.99 | 0.98, 1.01 | 0.582 | 1.00 | 0.98, 1.01 | 0.610 | 0.99 | 0.97, 1.01 | 0.380 | 1.00 | 0.97, 1.01 | 0.589 |
| NT-proBNP |  |  |  |  |  |  | 1.00 | 1.00, 1.00 | 0.210 | 1.00 | 1.00, 1.00 | 0.134 | 1.00 | 1.00, 1.00 | 0.238 | 1.00 | 1.00, 1.00 | 0.160 |
| LVEF |  |  |  |  |  |  | 0.96 | 0.93, 1.00 | 0.030 | 0.97 | 0.93, 1.00 | 0.051 | 0.97 | 0.94, 1.00 | 0.076 | 0.96 | 0.93, 1.00 | 0.028 |
| BMI |  |  |  |  |  |  |  |  |  | 1.04 | 0.99, 1.09 | 0.166 |  |  |  |  |  |  |
| IL-6 |  |  |  |  |  |  |  |  |  |  |  |  | 1.02 | 1.00, 1.04 | 0.053 |  |  |  |
| HbA1c |  |  |  |  |  |  |  |  |  |  |  |  |  |  |  | 1.01 | 0.99, 1.04 | 0.189 |
| **Cardiovascular Mortality** | | | | | | | | | |  |  |  |  |  |  |  |  |  |
| GPC-4 | 2.10 | 1.58, 2.79 | <0.001 | 1.93 | 1.45, 2.57 | <0.001 | 1.61 | 1.01, 2.56 | 0.045 |  |  |  |  |  |  |  |  |  |
| Age |  |  |  | 1.03 | 0.98, 1.08 | 0.264 | 1.03 | 0.97, 1.09 | 0.373 |  |  |  |  |  |  |  |  |  |
| Sex, female |  |  |  | 0.23 | 0.03, 1.75 | 0.157 | 0.22 | 0.03, 1.76 | 0.154 |  |  |  |  |  |  |  |  |  |
| eGFR |  |  |  |  |  |  | 0.96 | 0.92, 1.00 | 0.040 |  |  |  |  |  |  |  |  |  |
| NT-proBNP |  |  |  |  |  |  | 1.00 | 1.00, 1.00 | 0.520 |  |  |  |  |  |  |  |  |  |
| LVEF |  |  |  |  |  |  | 0.90 | 0.83, 0.97 | 0.004 |  |  |  |  |  |  |  |  |  |
| **Worsening heart failure hospitalization** | | | | | | | | | |  |  |  |  |  |  |  |  |  |
| GPC-4 | 1.57 | 1.28, 1.94 | <0.001 | 1.50 | 1.21, 1.86 | <0.001 | 1.15 | 0.75, 1.76 | 0.515 |  |  |  |  |  |  |  |  |  |
| Age |  |  |  | 1.02 | 0.99, 1.05 | 0.118 | 1.02 | 0.99, 1.06 | 0.244 |  |  |  |  |  |  |  |  |  |
| Sex, female |  |  |  | 0.78 | 0.36, 1.70 | 0.533 | 0.76 | 0.35, 1.67 | 0.498 |  |  |  |  |  |  |  |  |  |
| eGFR |  |  |  |  |  |  | 1.00 | 0.98, 1.02 | 0.793 |  |  |  |  |  |  |  |  |  |
| NT-proBNP |  |  |  |  |  |  | 1.00 | 1.00, 1.00 | <0.001 |  |  |  |  |  |  |  |  |  |
| LVEF |  |  |  |  |  |  | 0.97 | 0.93, 1.00 | 0.053 |  |  |  |  |  |  |  |  |  |

Results from Cox proportional hazards regression analyses, including competing risk analysis according to the Fine and Gray model. Presented as hazard ratios (HR) and 95% confidence intervals (CI) per one standard deviation (SD) increase of glypican-4. Multivariable models adjusted for age, sex (Model 1), additionally for eGFR, NT-proBNP, and LVEF (Model 2), and further adjusted for either BMI (Model 3), or IL-6 (Model 4), or HbA1c (Model 5). Hazard ratios (HR) and 95% confidence intervals (CI) per one standard deviation (SD) increase of glypican-4. *HFrEF, heart failure with reduced ejection fraction; GPC-4, glypican-4;* *eGFR, estimated glomerular filtration rate; NT-proBNP, N-terminal pro-brain natriuretic peptide; LVEF, left ventricular ejection fraction; BMI, body mass index; IL-6, interleukin 6; HbA1c, haemoglobin A1c.*

**Supplementary Table S2.** Cox proportional hazard analyses in the ATTR-CM cohort.

|  | **univariable** | | | **Model 1** | | | **Model 2** | | | **Model 3** | | | **Model 4** | | | **Model 5** | | |
| --- | --- | --- | --- | --- | --- | --- | --- | --- | --- | --- | --- | --- | --- | --- | --- | --- | --- | --- |
|  | **HR** | **95%CI** | **p** | **HR** | **95%CI** | **p** | **HR** | **95%CI** | **p** | **HR** | **95%CI** | **p** | **HR** | **95%CI** | **p** | **HR** | **95%CI** | **p** |
| **All-cause mortality** | | | | | | | | | |  |  |  |  |  |  |  |  |  |
| GPC-4 | 2.05 | 1.47, 2.86 | <0.001 | 2.15 | 1.48, 3.12 | <0.001 | 1.96 | 1.12, 3.44 | 0.018 | 1.93 | 1.05, 3.57 | 0.035 | 2.04 | 1.15, 3.63 | 0.013 | 1.96 | 1.13, 3.41 | 0.016 |
| Age |  |  |  | 1.01 | 0.93, 1.09 | 0.874 | 1.02 | 0.94, 1.10 | 0.701 | 1.02 | 0.93, 1.12 | 0.639 | 1.04 | 0.95, 1.14 | 0.403 | 1.03 | 0.94, 1.14 | 0.502 |
| Sex, female |  |  |  | 0.26 | 0.11, 0.66 | 0.010 | 0.17 | 0.06, 0.48 | 0.003 | 0.18 | 0.06, 0.54 | 0.007 | 0.21 | 0.07, 0.68 | 0.017 | 0.28 | 0.08, 0.95 | 0.058 |
| eGFR |  |  |  |  |  |  | 1.01 | 0.98, 1.04 | 0.587 | 1.01 | 0.98, 1.04 | 0.575 | 1.01 | 0.97, 1.04 | 0.740 | 1.01 | 0.97, 1.04 | 0.743 |
| NT-proBNP |  |  |  |  |  |  | 1.00 | 1.00, 1.00 | 0.166 | 1.00 | 1.00, 1.00 | 0.191 | 1.00 | 1.00, 1.00 | 0.130 | 1.00 | 1.00, 1.00 | 0.191 |
| LVEF |  |  |  |  |  |  | 0.98 | 0.94, 1.02 | 0.345 | 0.98 | 0.93, 1.02 | 0.311 | 0.98 | 0.93, 1.03 | 0.369 | 0.99 | 0.94, 1.04 | 0.630 |
| BMI |  |  |  |  |  |  |  |  |  | 0.94 | 0.81, 1.10 | 0.453 |  |  |  |  |  |  |
| IL-6 |  |  |  |  |  |  |  |  |  |  |  |  | 0.98 | 0.94, 1.02 | 0.314 |  |  |  |
| HbA1c |  |  |  |  |  |  |  |  |  |  |  |  |  |  |  | 1.02 | 0.98, 1.06 | 0.307 |
| **Cardiovascular Mortality** | | | | | | | | | |  |  |  |  |  |  |  |  |  |
| GPC-4 | 1.49 | 0.81, 2.72 | 0.198 |  |  |  |  |  |  |  |  |  |  |  |  |  |  |  |
| **Worsening heart failure hospitalization** | | | | | | | | | |  |  |  |  |  |  |  |  |  |
| GPC-4 | 1.65 | 1.22, 2.23 | 0.001 | 1.69 | 1.20, 2.38 | 0.003 | 0.89 | 0.50, 1.58 | 0.681 |  |  |  |  |  |  |  |  |  |
| Age |  |  |  | 0.99 | 0.93, 1.06 | 0.856 | 1.01 | 0.93, 1.10 | 0.740 |  |  |  |  |  |  |  |  |  |
| Sex, female |  |  |  | 0.48 | 0.19, 1.22 | 0.123 | 0.30 | 0.11, 0.82 | 0.019 |  |  |  |  |  |  |  |  |  |
| eGFR |  |  |  |  |  |  | 0.98 | 0.96, 1.00 | 0.110 |  |  |  |  |  |  |  |  |  |
| NT-proBNP |  |  |  |  |  |  | 1.00 | 1.00, 1.00 | 0.041 |  |  |  |  |  |  |  |  |  |
| LVEF |  |  |  |  |  |  | 0.95 | 0.91, 1.00 | 0.033 |  |  |  |  |  |  |  |  |  |

Results from Cox proportional hazards regression analyses, including competing risk analysis according to the Fine and Gray model. Presented as hazard ratios (HR) and 95% confidence intervals (CI) per one standard deviation (SD) increase of glypican-4. Multivariable models adjusted for age, sex (Model 1), additionally for eGFR, NT-proBNP, and LVEF (Model 2), and further adjusted for either BMI (Model 3), or IL-6 (Model 4), or HbA1c (Model 5). Hazard ratios (HR) and 95% confidence intervals (CI) per one standard deviation (SD) increase of glypican-4. *ATTR-CM, transthyretin amyloid cardiomyopathy; GPC-4, glypican-4;* *eGFR, estimated glomerular filtration rate; NT-proBNP, N-terminal pro-brain natriuretic peptide; LVEF, left ventricular ejection fraction; BMI, body mass index; IL-6, interleukin 6; HbA1c, haemoglobin A1c.*

**Supplementary Table S3.** Supposed cut-off values from area under the receiver operating characteristic curve analyses.

|  | **All-cause mortality** | **Cardiovascular Mortality** | **Worsening heart failure hospitalization** |
| --- | --- | --- | --- |
| **HFrEF cohort** |  |  |  |
| AUC | 0.73 | 0.78 | 0.72 |
| 95 % CI | 0.65, 0.81 | 0.65, 0.90 | 0.63, 0.80 |
| Optimal cut-off value (pg/mL) | 1630 | 1630 | 1630 |
| Sensitivity | 0.741 | 0.833 | 0.717 |
| Specificity | 0.660 | 0.583 | 0.623 |
| **ATTR-CM cohort** |  |  |  |
| AUC | 0.66 | 0.56 | 0.72 |
| 95 % CI | 0.54, 0.77 | 0.36, 0.75 | 0.63, 0.81 |
| Optimal cut-off value (pg/mL) | 2774 | 2774 | 2774 |
| Sensitivity | 0.563 | 0.500 | 0.471 |
| Specificity | 0.787 | 0.716 | 0.759 |

Optimal cut-off values of glypican-4 to predict all-cause mortality, cardiovascular mortality, and
worsening heart failure hospitalization. *HFrEF,* *heart failure with reduced ejection fraction;
ATTR-CM, transthyretin amyloid cardiomyopathy; AUC, area under the curve; CI, confidence interval.*


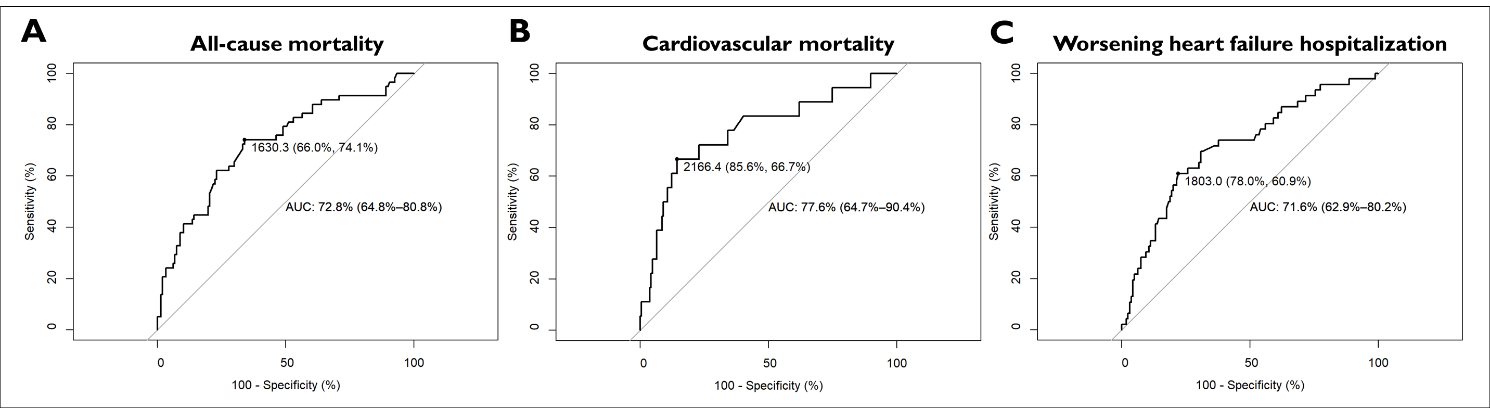
**Supplementary Figure S1.** Receiver operating characteristic curve analyses in the HFrEF cohort.

(A) ROC-AUC analysis for all-cause mortality, (B) cardiovascular mortality, and (C) worsening heart failure hospitalization including the AUC (95% CI) and the proposed cutt-off value (specifity, sensitivity). *HFrEF, heart failure with reduced ejection fraction; ROC, receiver operating characteristic; AUC, area under the curve; CI, confidence interval.*


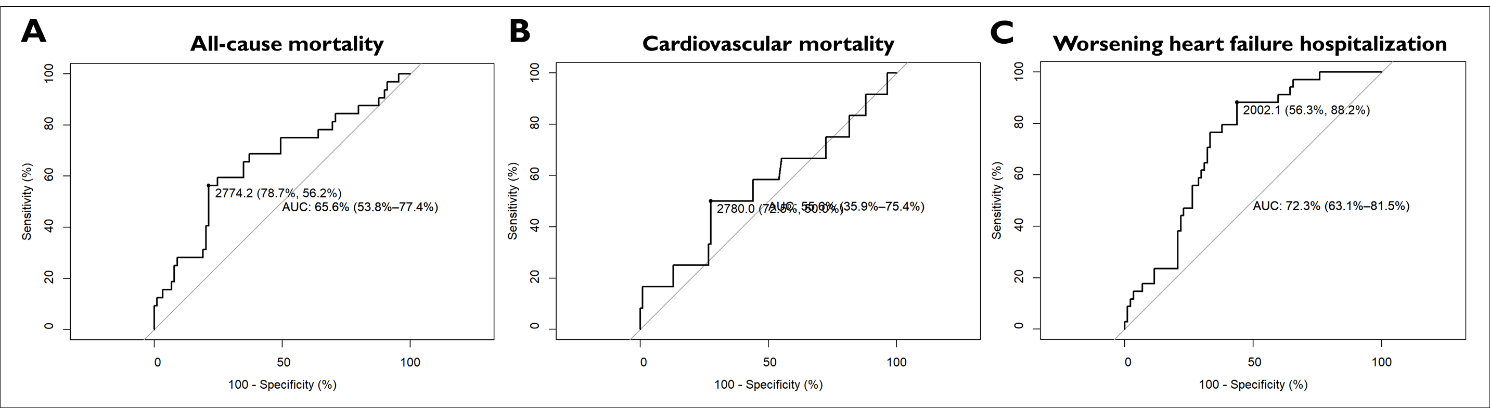
**Supplementary Figure S2.** Receiver operating characteristic curve analyses in the ATTR-CM cohort.

(A) ROC-AUC analysis for all-cause mortality, (B) cardiovascular mortality, and (C) worsening heart failure hospitalization including the AUC (95% CI) and the proposed cutt-off value (specifity, sensitivity). *ATTR-CM, transthyretin amyloid cardiomyopathy*; *ROC, receiver operating characteristic; AUC, area under the curve; CI, confidence interval.*
